# Supplementary material for: Comparison of deep learning models in automatic classification of coffee bean species
Source: PeerJ Comput Sci. 2025 Apr 7;11:e2759. doi: 10.7717/peerj-cs.2759 (PMC12190548; doi:10.7717/peerj-cs.2759)
Supplement: Supplemental Information 2 [file peerj-cs-11-2759-s002.docx]

**IMAGE CLASSIFICATION CODE**

This Python code is designed to classify images using deep learning models on a pre-defined dataset of coffee images. It involves preparing the dataset for testing, loading the images, generating the test data pipeline, and providing an evaluation setup for multiple deep learning models. The code also computes various classification metrics to evaluate model performance.

**Code Overview**

The main purposes of the code are:

1. *Dataset Preparation:* Create a DataFrame containing paths and labels of images in the test dataset.
2. *Data Generation:* Use Keras' ImageDataGenerator to load and preprocess images for evaluation.
3. *Model Evaluation:* Set up the framework to load different deep learning models and evaluate their performance using standard classification metrics.

**Step-by-Step Implementation**

***Step 1: Import Libraries***

import os

import numpy as np

import pandas as pd

import matplotlib.pyplot as plt

import seaborn as sns

from sklearn.metrics import classification_report, confusion_matrix, accuracy_score

import tensorflow as tf

from tensorflow.keras.preprocessing.image import ImageDataGenerator

**Explanation:** Necessary libraries are imported. These include libraries for data manipulation (pandas, numpy), visualization (matplotlib, seaborn), model evaluation (sklearn), and TensorFlow for deep learning functionality.

***Step 2: Define test_df Function to Generate DataFrame***

def test_df(ts_path):

    classes = []

    class_paths = []

    files = os.listdir(ts_path)

    for file in files:

        label_dir = os.path.join(ts_path, file)

        label = os.listdir(label_dir)

        for image in label:

            image_path = os.path.join(label_dir, image)

            class_paths.append(image_path)

            classes.append(file)

    image_classes = pd.Series(classes, name='Class')

    image_paths = pd.Series(class_paths, name='Class Path')

    ts_df = pd.concat([image_paths, image_classes], axis=1)

    return ts_df

**Explanation:** This function takes a directory path (ts_path) as input, which contains subdirectories of images for each class. It then iterates through each image file to collect image paths and class labels, returning a DataFrame that lists each image's path and class.

***Step 3: Load the Test Data***

ts_df = test_df('…/Python/Coffee_Image_Dataset_Split/test')

**Explanation:** The function test_df is called with the path to the test dataset, generating a DataFrame (ts_df) containing image paths and their corresponding classes.

***Step 4: Define and Configure the Data Generator***

img_size = (224, 224)

gen = ImageDataGenerator()

ts_gen = gen.flow_from_dataframe(ts_df, x_col='Class Path', y_col='Class', batch_size=16, target_size=img_size, shuffle=False)

**Explanation:** Using ImageDataGenerator, a data generator (ts_gen) is created to preprocess images by resizing them to 224x224 pixels. The generator reads the DataFrame and outputs batches of images with their corresponding labels, which are used for model evaluation.

***Step 5: Define Class Labels***

class_dict = ts_gen.class_indices

classes = list(class_dict.keys())

num_classes = len(classes)

**Explanation:** Here, class indices are extracted from the data generator (class_dict), providing a dictionary of class names and their corresponding labels. This step is essential for interpreting the classification results.

***Step 6: Define Models to Load and Evaluate***

model_name = ['Xception', 'DenseNet201', 'InceptionResNetV2', 'DenseNet121', 'InceptionV3']

**Explanation:** This list of model names serves as a reference for the models to be loaded and evaluated. Each model will be evaluated independently, allowing comparison of results across different architectures.

***Step 7: Load and Evaluate Models***

for model_name, model_path in model_paths.items():

    print(f'\nLoading and evaluating model: {model_name}')

    model = load_model(model_path)

    print(f'\nEvaluating model: {model_name}')

    eval_result = model.evaluate(ts_gen, verbose=1)

    print(f'Model {model_name} test loss: {eval_result[0]:.4f}, test accuracy: {eval_result[1]:.4f}')

**Explanation:** In this loop, each model in model_paths is loaded and evaluated.

- *Model Loading:* load_model(model_path) loads the model from the specified path in model_paths, a dictionary where keys are model names and values are file paths to the saved models.
- *Model Evaluation:* Each model’s performance is assessed on the test data (ts_gen). The evaluation function outputs the test loss and accuracy, which are printed for each model.

***Step 8: Generate Classification Reports***

for model_name, model_path in model_paths.items():

    print(f'\nLoading and evaluating model: {model_name}')

    model = load_model(model_path)

    # Predictions

    pred = model.predict(ts_gen)

    pred_classes = np.argmax(pred, axis=1)

    labels = dict((v, k) for k, v in class_dict.items())

    pred2 = [labels[k] for k in pred_classes]

    y_test = ts_df['Class']

    # Classification report

    print(f'\nClassification Report for {model_name}:\n')

    print(classification_report(y_test, pred2))

**Explanation:**

***Model Loading:*** Similar to Step 7, each model in model_paths is loaded using load_model(model_path).

***Predictions:*** After loading, each model makes predictions on the test data (ts_gen). model.predict(ts_gen) produces probabilities for each class, and np.argmax(pred, axis=1) converts these probabilities into predicted class indices.

***Label Mapping:*** The predicted indices are then mapped to class names (pred2) based on class_dict to match the format of the true labels.

***Classification Report:*** The classification_report function compares the true labels (y_test) with the predicted labels (pred2), generating precision, recall, f1-score, and support for each class, which is then printed.

***Step 9: Generate and Visualize the Confusion Matrix***

for model_name, model_path in model_paths.items():

    print(f'\nLoading and evaluating model: {model_name}')

    model = load_model(model_path)

    # Predictions

    pred = model.predict(ts_gen)

    pred_classes = np.argmax(pred, axis=1)

    labels = dict((v, k) for k, v in class_dict.items())

    pred2 = [labels[k] for k in pred_classes]

    y_test = ts_df['Class']

    cm = confusion_matrix(y_test, pred2)

    plt.figure(figsize=(10, 7))

    sns.heatmap(cm, annot=True, fmt='g', vmin=0, cmap='Blues')

    plt.xticks(ticks=[i + 0.5 for i in range(len(classes))], labels=classes, rotation=45)

    plt.yticks(ticks=[i + 0.5 for i in range(len(classes))], labels=classes, rotation=0)

    plt.xlabel("Predicted")

    plt.ylabel("Actual")

    plt.title(f"{model_name} Confusion Matrix")

    plt.show()

**Explanation:**

*Predictions:* As in the previous steps, each model is loaded and used to make predictions on the test data.

*Confusion Matrix:* confusion_matrix(y_test, pred2) generates a confusion matrix comparing the true labels (y_test) with predicted labels (pred2).

*Visualization:* Using seaborn.heatmap, the confusion matrix is visualized as a heatmap for better interpretability.

- The x-axis shows predicted classes, and the y-axis shows actual classes.
- Annotations display the count of predictions for each actual-predicted class pair.
- This helps identify which classes the model confuses, offering insights into potential areas for model improvement.

***Step 10: Calculate and Plot the ROC AUC Curve***

for model_name, model_path in model_paths.items():

    print(f'\nLoading and evaluating model: {model_name}')

    model = load_model(model_path)

    # Predictions

    pred = model.predict(ts_gen)

    if len(classes) == 2:

        roc_auc = roc_auc_score(y_test.map(class_dict), pred[:, 1])

        print(f'ROC AUC for {model_name}: {roc_auc:.4f}')

        fpr, tpr, _ = roc_curve(y_test.map(class_dict), pred[:, 1])

        plt.figure(figsize=(8, 6))

        plt.plot(fpr, tpr, color='darkorange', lw=2, label=f'ROC curve (area = {roc_auc:.4f})')

        plt.plot([0, 1], [0, 1], color='navy', lw=2, linestyle='--')

        plt.xlim([0.0, 1.0])

        plt.ylim([0.0, 1.05])

        plt.xlabel('False Positive Rate')

        plt.ylabel('True Positive Rate')

        plt.title(f'{model_name} Receiver Operating Characteristic')

        plt.legend(loc="lower right")

        plt.show()

**Explanation:**

*Binary Classification Check:* The code first checks if the problem is binary (len(classes) == 2). ROC AUC is only applicable to binary classification or one-vs-rest multiclass setups.

*ROC AUC Calculation:* Using roc_auc_score, the code calculates the ROC AUC score for the binary classifier by mapping the true labels (y_test) to class indices (class_dict) and comparing them with model predictions (pred[:, 1]), representing the probability of the positive class.

*ROC Curve Plotting:* The code then computes the False Positive Rate (fpr) and True Positive Rate (tpr) to plot the ROC curve. A diagonal line (baseline) represents random performance, while the ROC curve shows the model’s ability to distinguish between classes. A higher area under the curve indicates better performance.
